# Subcortical volume reduction and cortical thinning 3 months after switching to clozapine in treatment resistant schizophrenia

**DOI:** 10.1038/s41537-022-00230-2

**Published:** 2022-03-02

**Authors:** Fanni Krajner, Laila Hadaya, Grant McQueen, Kyra-Verena Sendt, Amy Gillespie, Alessia Avila, John Lally, Emily P. Hedges, Kelly Diederen, Oliver D. Howes, Gareth J. Barker, David J. Lythgoe, Matthew J. Kempton, Philip McGuire, James H. MacCabe, Alice Egerton

**Affiliations:** 1 Department of Psychosis Studies, Institute of Psychiatry, Psychology & Neuroscience, King’s College London, De Crespigny Park, London, UK; 2 Department of Psychiatry, University of Oxford, Warneford Hospital, Oxford, UK; 3 Department of Psychiatry, Royal College of Surgeons in Ireland, Dublin, Ireland; 4 South London and Maudsley NHS Trust, London, UK; 5 Department of Neuroimaging, Centre for Neuroimaging Sciences, Institute of Psychiatry, Psychology & Neuroscience, King’s College London, De Crespigny Park, London, UK

**Keywords:** Schizophrenia, Biomarkers, Schizophrenia

## Abstract

The neurobiological effects of clozapine are under characterised. We examined the effects clozapine treatment on subcortical volume and cortical thickness and investigated whether macrostructural changes were linked to alterations in glutamate or N-acetylaspartate (NAA). Data were acquired in 24 patients with treatment-resistant schizophrenia before and 12 weeks after switching to clozapine. During clozapine treatment we observed reductions in caudate and putamen volume, lateral ventricle enlargement (*P* < 0.001), and reductions in thickness of the left inferior temporal cortex, left caudal middle frontal cortex, and the right temporal pole. Reductions in right caudate volume were associated with local reductions in NAA (*P* = 0.002). None of the morphometric changes were associated with changes in glutamate levels. These results indicate that clozapine treatment is associated with subcortical volume loss and cortical thinning and that at least some of these effects are linked to changes in neuronal or metabolic integrity.

## Introduction

Clozapine is the only antipsychotic licenced for treatment-resistant schizophrenia (TRS)^1^ but the neurobiological processes underlying its superior efficacy are unknown. TRS is associated with more pronounced brain structural abnormalities than treatment responsive illness^2,3^ and clozapine may have some neuroprotective effects^4,5^. Recently, widespread cortical thinning and reductions in subcortical volume have been reported 6–9 months after clozapine initiation^6,7^. However, the underlying mechanisms are poorly understood.

The most robustly described brain macrostructural features of schizophrenia include volumetric reductions in the hippocampus, amygdala, thalamus, and accumbens, enlargement of the lateral ventricles, and widespread cortical thinning^8–12^. These changes occur at or before psychosis onset^13–18^ and progression may be most rapid at the earlier stages of illness^19–24^. In addition to illness mechanisms, antipsychotic administration could also contribute to grey matter volume reductions. The extent of antipsychotic exposure is positively associated with cortical grey matter loss, reduction in whole-brain grey matter volume and lateral ventricle enlargement^11,25–31^, and in animal models antipsychotic administration reduces cortical and total grey and white matter volume^32–34^. Importantly, however, grey matter decreases during antipsychotic treatment occur in parallel with clinical improvement, and it is unclear whether these macrostructural changes reflect underlying neurotoxic or neuroadaptive processes, or some combination^35,36^.

Compared to patients who have shown a good response to antipsychotic treatment, a greater degree of reduction in cortical volume and thickness may be present in patients who have not responded well, including those meeting criteria for TRS and being prescribed clozapine^2,3,31,37–41^. However, it is difficult to determine from cross-sectional studies whether the greater magnitude of cortical grey matter loss and thinning observed in people with TRS^2,3,37–41^ primarily reflects the existence of a neurobiologically distinct TRS illness subtype, a general effect of illness severity, greater prior antipsychotic medication burden, or the neurobiological consequences of clozapine treatment.

Only a few longitudinal studies which have examined brain macrostructural changes on switching to clozapine. The most robust finding is a reduction in caudate volume, which has been observed after 6 months to 2 years of clozapine treatment^7,42–45^. Subcortical volume reductions during clozapine treatment may also extend to the putamen, thalamus, and hippocampus and occur in parallel with enlargement of the lateral ventricles^7^. The effects of clozapine on cortical structure are less clear. Widespread cortical thinning has been reported 6–9 months after switching to clozapine^6^, as have slight decreases in cortical thickness in the left pars triangularis and opercularis and left caudal middle frontal areas over 2 years of clozapine or risperidone treatment in first episode psychosis^46^. Over a 5 year period, cortical thinning was also more pronounced in patients who had received clozapine during the interscan interval than in those who had not taken clozapine^47^, and, in childhood onset schizophrenia, thinning within a small dorsolateral prefrontal cortical area was more marked in patients taking clozapine than those taking olanzapine^48^. Conversely, increases in cortical grey matter over 26 months of clozapine treatment have also been reported^49^, and frontal grey matter and cortical thinning may be less pronounced after treatment with second-generation antipsychotics including clozapine compared to treatment with first-generation antipsychotics^11,23,28,47,50^. Whilst these longitudinal studies indicate clozapine has effects on brain structure, all the studies were conducted after 6 months or more of clozapine treatment. However, the clinical effects of clozapine are seen within 6–12 weeks^51,52^. Thus, it remains unclear if structural changes associated with clozapine are linked to its therapeutic effects or occur later.

There are several interacting biological mechanisms that may underlie the observed macrostructural changes in schizophrenia and during treatment with antipsychotics, including clozapine. Macrostructural deficits in schizophrenia are thought to principally reflect apoptotic loss of synapses and dendrites resulting from glutamate excitotoxicity^53,54^ or neuroinflammation and microglial activation^14,55^, rather than neuronal loss^56–58^. Consistent with the expected effect of glutamate excitotoxicity, studies combining structural imaging with proton magnetic resonance spectroscopy (^1^H-MRS) to measure regional glutamatergic metabolites find anterior cingulate cortex (ACC) glutamatergic metabolite levels are negatively associated with cortical thickness or volume^41,59^ and caudate Glx (the sum of glutamate and its metabolite glutamine) is negatively associated with caudate volume in first episode psychosis^60^. ACC glutamatergic metabolites may be particularly elevated in the TRS illness subtype^61–65^ and clozapine reduces brain glutamate levels in animal models^66–68^. In humans, we have recently shown that glutamate is decreased in the caudate, but not in the ACC, 12 weeks after patients with TRS were switched to clozapine treatment^69^. The macrostructural changes observed during clozapine treatment^6,7,42–46,49^ could therefore have a glutamatergic basis. Clozapine may also be able to promote neurogenesis^4^, increase dendritic spine density^70,71^, reduce microglial activation and promote remyelination^72,73^, neurochemical plasticity^74^, and induce non-lethal apoptotic activity in the cortex via caspase-3^75^. While it is unclear how these cellular and microstructural effects translate to macrostructural changes observed in vivo with structural MRI, N-acetyl aspartate (NAA), a further metabolite detectable with ^1^H-MRS, provides a marker of neuronal and metabolic integrity ^76–79^.

With an overall aim of better characterising the neurobiological processes that occur during clozapine treatment, the current study examined changes in subcortical volume and cortical thickness in patients with TRS over 12 weeks after switching to clozapine treatment. Based on previous reports, we hypothesised that over this period we would observe reductions in caudate^7,42–45^, putamen, thalamus and hippocampus volumes, lateral ventricular enlargement^7^, and cortical thinning^6,46^. We also hypothesised that reductions in caudate volume during clozapine treatment would be associated with local reductions in glutamate and NAA^69^, and that cortical thinning would associate with changes in ACC glutamate and NAA levels. As exploratory analyses we tested for associations between structural changes and symptomatic improvement over the 12-week observation period.

## Results

### Sample characteristics

After QC, structural data for analyses were available in 24 patients at both baseline and after 12 weeks of clozapine treatment (Table 1). The demographic and clinical characteristics of the patient sample are provided in further detail in our previous publication^69^. Co-medication included antidepressant medication in 7 patients (including citalopram, sertraline, venlafaxine, escitalopram, and fluoxetine) and GABAergic medication in 9 patients (including clonazepam lorazepam, chlordiazepoxide, valproate, lithium, and zopiclone). Symptom severity and functioning significantly improved over clozapine treatment (all *P* < 0.01; Table 1).

**Table 1:** Characteristics of the sample.

Age	38.62 ± 12.81
Sex (M/F)	18/6
Age of onset	26.29 ± 8.77
Duration of illness	13.58 ± 8.62
Previous antipsychotic trials (median; Q1; Q3)	3; 2; 5
Previous clozapine trial (Y/N)	4/20
Number of hospitalisations (median; Q1; Q3)	3; 1; 5
Antipsychotic prior to clozapine initiation (N):	
Olanzapine	5
Aripiprazole	5
Quetiapine	4
Amisulpride	3
Risperidone	2
Flupentixol	2
Haloperidol	1
Zuclopenthixol	1
Paliperidone	1
Antidepressant medication: N (Y/N)	7/17
GABAergic medication: N (Y/N)	9/15
Current tobacco use N (Y/N)	8/16
Current alcohol use N (Y/N)	4/20
Current cannabis use N, (Y/N)	3/21
Baseline PANSS Positive	18.04 ± 5.83
12-week PANSS Positive	13.75 ± 4.73*
Baseline PANSS Negative	19.17 ± 7.63
12-week PANSS Negative	15.88 ± 6.13*
Baseline PANSS General	34.25 ± 7.10
12-week PANSS General	26.54 ± 5.32*
Baseline PANSS Total	71.46 ± 15.85
12-week PANSS Total	56.21 ± 13.37*
Baseline GAF	46.96 ± 10.80
12-week GAF	59.38 ± 8.66*
12-week clozapine plasma level ng/mL	481.8 ± 307.7
12-week clozapine dose	335.9 ± 129.9
eTIV	1473915 ± 136938
SPC in caudate Glu_corr_	−37.91 ± 62.19
SPC in caudate NAA_corr_	−32.29 ± 60.94
SPC ACC Glu_corr_	−0.34 ± 98.01
SPC in ACC NAA_corr_	14.25 ± 79.35

Values are provided as mean ± standard deviation unless otherwise stated. Abbreviations: *ACC* Anterior cingulate cortex; *eTIV* Estimated total intracranial volume; *GAF* Global Assessment of Functioning; *Glu*_*corr*_ Glutamate corrected for voxel tissue composition; *NAA*_*corr*_ N-acetylaspartate corrected for voxel tissue composition; *PANSS* Positive and Negative Syndrome Scale; *SPC* Symmetrized Percentage Change. * *P* < 0.01 follow-up compared to baseline.

### Change in subcortical volume over 12 weeks of clozapine treatment

**Fig. 1:**
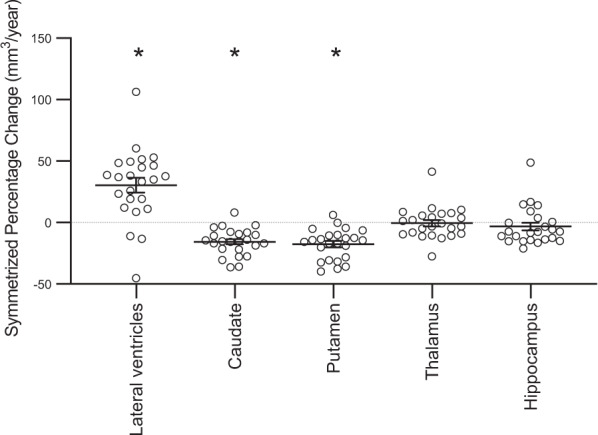
Symmetrized percentage change (SPC) in subcortical volumes 12 weeks after switching to clozapine treatment. The graph presents the individual data points, together with the mean and standard error of the mean for each volume. Increases in lateral ventricle volume and decreases in caudate and putamen volume significantly differed from zero (* One sample T-test; *P* < 0.01). Please note SPC values are 4–5 fold greater than the percentage change (Table 2).

Volumes of subcortical structures before and 12 weeks after switching to clozapine treatment are provided in Table 2. Over this period, lateral ventricle volume significantly increased and the volume of the caudate and putamen significantly decreased (Table 2 and Fig. 1). No significant change was observed in hippocampus or thalamus volume (Table 2 and Fig. 1). Sensitivity analyses did not alter these findings. Age and eTIV were not significantly associated with SPC in subcortical volume (all *P* > 0.05). In the caudate and putamen, volumetric reduction was more marked in male compared to female participants (caudate: mean ± s.d. male: −19.08 ± 9.43; female: −4.98 ± 7.67; *T*_22_ = 3.30; *P* = 0.003; putamen male: −20.36 ± 12.65; female: −9.08 ± 5.90; *T*_22_ = 2.09; *P* = 0.049). Subcortical volumetric changes were not significantly associated with plasma clozapine levels at 12 weeks or the percentage change in PANSS Total score (*P* > 0.05 Supplement Fig. 1).

**Table 2:** Subcortical volumes at before and 12 weeks after clozapine initiation.

	Volume baseline (mm^3^)	Volume 12 weeks (mm^3^)	PC	SPC (mm^3^/year)	Statistic
Lateral ventricles	16633; Q1: 10713; Q3: 30120	16838; Q1: 12592; Q3: 31855	7.50 ± 7.27	30.44 ± 29.38*	*T*_23_ = 5.07; *P* < 0.001; *d* = 1.04
Caudate	7945; Q1: 7000; Q3: 9310	7552; Q1: 6687; Q3: 8990	−3.49 ± 2.40	−15.56 ± 10.83*	*T*_23_ = 7.04; *P* < 0.001; *d* = 1.44
Putamen	11198 ± 1274	10758 ± 1269	−3.92 ± 2.71	−17.54 ± 12.28*	*T*_23_ = 7.00; *P* < 0.001; *d* = 1.43
Thalamus	12962 ± 1645	12939 ± 1549	−0.45; −2.14; 1.59	−1.97; −9.30; 6.51	*N* = 24; *z* = 0.66; *P* = 0.51
Hippocampus	7797 ± 694	7735 ± 629	−1.72; −3.21; 0.70	−7.55; −14.06; 2.20	*N* = 24; *z* = 1.6; *P* = 0.11

The table presents data in 24 patients with treatment-resistant schizophrenia who completed MRI at both timepoints. Normally distributed data are expressed as mean ± standard deviation, non-normally distributed data are expressed as median; quartile 1 (Q1); quartile 3 (Q3). PC: Percentage volumetric change, calculated as (volume at follow-up − volume at baseline)/(volume at baseline)*100. The statistical significance of the symmetrized percentage change (SPC) was evaluated using one-sample T-tests for normally distributed data and one sample Wilcoxon signed rank test for non-normally distributed data. * Significant increases in lateral ventricle volume and decreases in caudate and putamen volume were observed over 12 weeks of clozapine treatment.

### Cortical thinning over 12 weeks of clozapine treatment

**Fig. 2:**
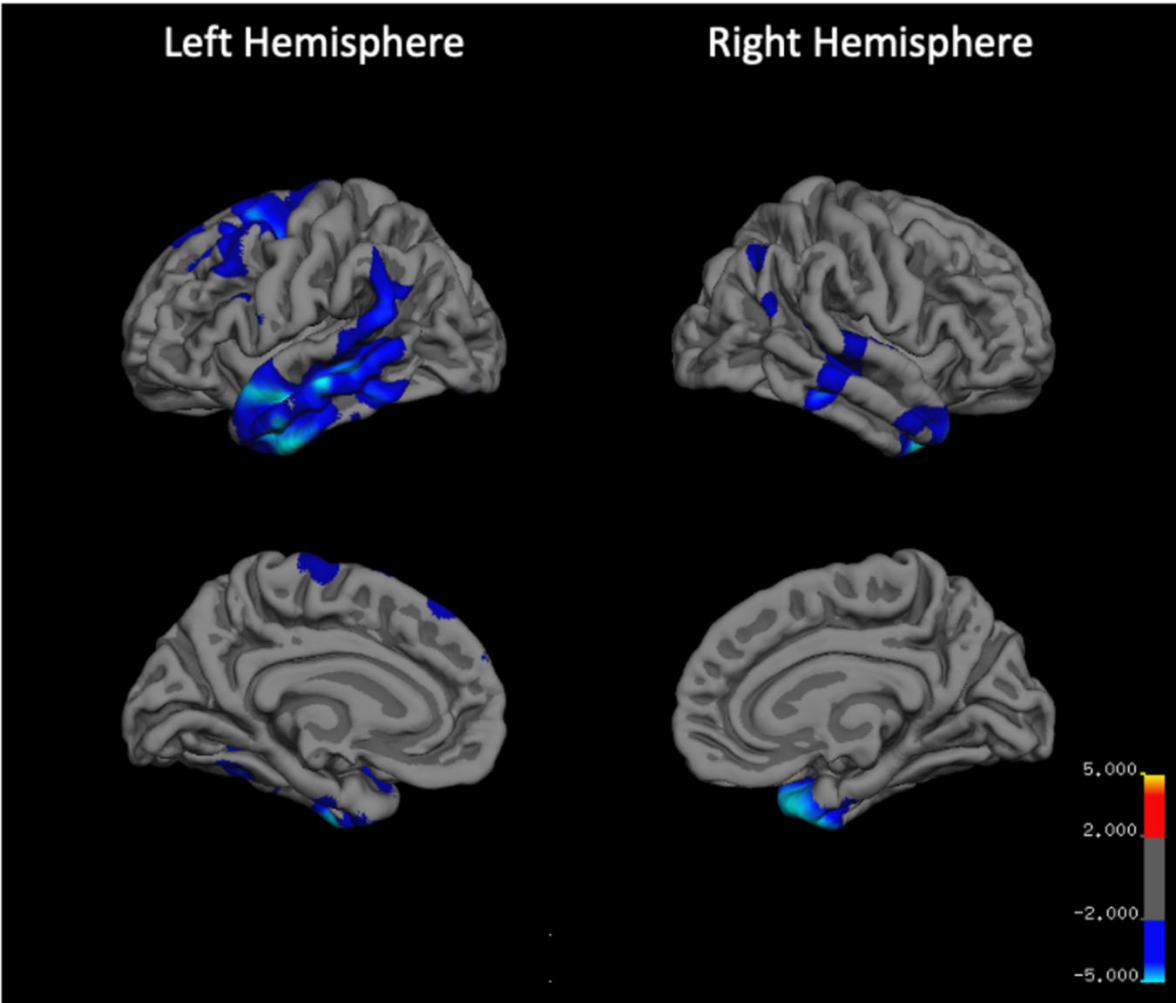
Cortical thinning over 12 weeks after switching to clozapine treatment. Cortical thinning was evaluated as the symmetrized percentage change. The significance in the display is the −log10 *P* value, where blue represents areas of significant reductions in cortical thickness after 12 weeks of clozapine treatment. No areas of significant increase in cortical thickness were detected.

Cortical thinning was apparent over 12 weeks of clozapine treatment (Fig. 2), reaching significance in three clusters situated in the left inferior temporal cortex, left caudal middle frontal cortex, and right temporal pole (Table 3). Within these regions, cortical thinning did not differ by sex, age, or eTIV, and was not significantly associated with the percentage improvement in PANSS total symptom score or with clozapine plasma levels at 12 weeks (all *P* > 0.05). No significant clusters relating to increases in cortical thickness over time were identified. Analyses examining the relationships between the three significant clusters of cortical thinning and reductions in subcortical volume indicated that thinning in the right temporal pole was associated with putamen volume reduction (Supplement Table 1, df = 21; *r* = 0.61; *P* = 0.002, controlling for sex).

**Table 3:** Clusters showing significant cortical thinning over 12 weeks of clozapine treatment.

Hemisphere	Annotation	Max	Size mm^2^	*X*; *Y*; *Z*	CWP	Extracted SPC mean ± s.d.
Left	Inferior temporal	−6.267	15260	−40.1; −6.1; −42.3	0.002	−13.95 ± 14.79
Left	Caudal middle frontal	−5.195	10710	−28.8; 5.2; 52.8	0.002	−11.57 ± 14.18
Right	Temporal pole	−6.496	9403	31.7; 6.2; −38.2	0.018	−23.83 ± 20.85

CWP Cluster-wise *P* value; *Max* maximum −log10 *P* value of the cluster. Size: cluster surface area in mm^2^; SPC symmetrized percentage change; extracted from the annotated region. *X*; *Y*; *Z*: Talairach coordinates of the maximum.

### Relationships with glutamate and NAA

In line with our previous report in the same cohort^69^, SPC of caudate Glu_corr_ and NAA_corr_ significantly differed from zero, with lower levels after 12 weeks of clozapine treatment (Glu_corr_: *T*_20_ = 3.04; *P* = 0.006; NAA_corr_: *T*_20_ = 2.43; *P* = 0.03; Table 1). There was no significant change in ACC SPC in Glu_corr_ and NAA_corr_ (*P* > 0.05, Table 1). The SPC of right caudate volume was positively associated with the SPC of right caudate NAA_corr_ (df = 18, *r* = 0.65; *P* = 0.002 controlling for sex, Supplement Fig. 2), but not with that of right caudate Glu_corr_ (*P* > 0.05, Supplement Fig. 2). The relationship between SPC of right caudate volume and NAA_corr_ remained present after removing two outlying values identified by Cook’s D (df = 16, *r* = 0.57; *P* = 0.01). Relationships between the SPC in ACC Glu_corr_ or NAA_corr_ and SPC in the three significant regions of cortical thinning were non-significant (all *P* > 0.05).

## Discussion

Our main finding is that in patients with TRS, 12 weeks of clozapine treatment was associated with longitudinal reductions in caudate and putamen volume and cortical thickness, and lateral ventricle enlargement. The relationship between the reductions in putamen volume and temporal pole thickness provided some indication that subcortical and cortical reductions may occur within the same individuals. The reductions in right caudate volume were also associated with local reductions in NAA. Although symptoms improved over the same period of clozapine administration, the structural changes were not associated with the clinical response to clozapine. Our findings of subcortical volumetric reduction, ventricular enlargement, and cortical thinning during clozapine treatment are generally consistent with previous observations over longer periods of clozapine administration^6,7,42–46^. Moreover they indicate that morphological changes begin within the first months of switching from non-clozapine antipsychotics to clozapine.

Previous studies reporting reductions in caudate volume^7,42–45^, putamen volume, and ventricular enlargement^7^ with clozapine have evaluated brain macrostructure over much longer treatment periods of 6 months to 2 years. The mean percentage changes in volume that we observed in these regions over 12 weeks of clozapine (caudate: −3.5; putamen: −4%; lateral ventricles: +7.5%) appear somewhat lower in magnitude than those observed by Tronchin et al^7^. over 6–9 months of clozapine (caudate: −4.8; putamen −6.1; lateral ventricles +15%)^7^. Tronchin et al^7^. also detected decreases in volume in the thalamus and hippocampus, regions which did not reach significance in our cohort. Qualitatively, the pattern of cortical thinning that we observed over 12 weeks clozapine also appears similar, although less widespread than that over longer periods of clozapine administration^6^. Overall, our findings considered together with previous research found that subcortical volume loss^7,42–45^ and cortical thinning^6,46^ occur during clozapine treatment and may indicate that these changes may emerge within the first months of clozapine treatment and further progresses over longer periods of administration. Treatment with non-clozapine antipsychotics is associated with increases in caudate and putamen volume^80–89^. Therefore, the observed decreases in caudate and putamen volume after switching to clozapine treatment could indicate a reversal of the volumetric increases associated with prior non-clozapine antipsychotic treatment and/or effects of discontinuing treatment with non-clozapine antipsychotics.

While we detected reductions in caudate, but not ACC, glutamate during clozapine treatment in the same patient cohort^69^, in the current study we found no evidence to implicate changes in glutamate levels with cortical thinning or caudate volume reduction. Instead, reductions in caudate volume during clozapine treatment were associated with local reductions in NAA, which may indicate decreases in the integrity of neurons or oxidative metabolism in mitochondria^76–79^. Volumetric decreases during antipsychotic treatment may reflect tissue remodelling in response to physiological attempts to meet increased metabolic demands^90^, as clozapine and other antipsychotics can impair mitochondrial function^91–94^. However, it remains unknown whether the observed volumetric reductions and cortical thinning during clozapine treatment reflect excitotoxicity-induced apoptosis^54^, increased metabolic stress^90^, or neuroadaptive processes including compensatory pruning of malfunctioning neurons^95^, reduced microglial activation, and demyelination^36,72,73^ or increases in subcortical white matter neuronal densities^74^. Future research could investigate these mechanisms further by combining structural MRI with indices of neuroinflammation, synaptic density^96^, peripheral mitochondrial activity, and central oxidative capacity^90^ during treatment with clozapine.

Unlike previous studies over longer periods of clozapine treatment^6,7,43^, we did not detect any significant associations between structural changes and symptomatic improvement, and it could be that these associations are only observed after longer periods of clozapine administration. In the study of Scheepers et al.^43^, while the most marked decreases in caudate volume occurred during the initial 6 months, only caudate reduction at 12 months was related to symptomatic improvement. Previous studies comparing structural changes in clozapine responder and clozapine non-responder groups have found either no significant difference in subcortical volume change^7^, or greater decreases in caudate volume^43^ and less cortical thinning^6^ in clozapine responders. One limitation of our study is that the modest sample size may have been underpowered to detect associations with continuous measures of clinical improvement. Alternatively, the lack of significant association with clinical measures could indicate that mechanisms independent of macrostructural changes primarily drive symptomatic improvement in the first months after clozapine treatment.

Strengths of our study include the longitudinal design, examining patients over a similar period of clozapine treatment using multimodal imaging. Application of the automated Freesurfer longitudinal pipeline, incorporating a within-subjects template and analysis of SPC increases reliability and sensitivity and removes asymmetry bias^97^. As we did not observe any significant associations between clozapine plasma levels and structural changes, and as our study design did not include a longitudinal comparison group of patients with TRS switched to an antipsychotic other than clozapine, this prohibits interpretation of the findings in relation to effects of clozapine treatment specifically versus on-going progressive illness processes. However, we consider it unlikely that the observed effects occurred independent of clozapine treatment, as most patients had already been unwell and treated with antipsychotics for several years prior to baseline, and as other studies have detected associations between subcortical volumetric reduction and clozapine levels^7^. Additionally, as our study did not include two scans in a comparison healthy volunteer sample, we are unable to evaluate the results in relation to within-subject repeatability. A further consideration is that structural MRI and ^1^H-MRS measurements including NAA reflect tissue water relaxation times, which could potentially be influenced by clozapine as well as other factors^90,98^, although antipsychotics have not been found to affect T1 or T2 relaxation times ^32,99^.

In conclusion, the reductions in the volume of the caudate and putamen, lateral ventricle enlargement, and cortical thinning over 12 weeks after switching clozapine treatment in patients with TRS are generally consistent with previous findings over longer periods of clozapine administration^6,7,42–46^, although they may be more limited in spatial extent^6,7^. While this adds further evidence that clozapine treatment is associated with subcortical volume loss and cortical thinning, importantly it remains unknown whether these macrostructural changes reflect harmful or neuroadaptive processes. The association between local reductions in NAA and caudate volume may implicate decreases in neuronal or metabolic activity. Future work combining structural MRI with measures of synaptic integrity, metabolic activity, and neuroinflammatory markers may further elucidate the underlying mechanisms.

## Methods

Inclusion required an ICD-10 diagnosis of schizophrenia or schizoaffective disorder, the presence of treatment-resistant illness, and being about to commence clozapine titration as part of normal clinical care^69^. Presence of TRS was inferred from medical records and discussion with the treating psychiatrist, with criteria including at least two previous trials of a non-clozapine antipsychotic within the recommended dose range for at least 6 weeks and referral for clozapine initiation^69^. Exclusion criteria included pregnancy or contraindication to MRI at 3 Tesla, drug dependency as defined in DSM-IV, or having been prescribed clozapine within the 3 months prior to the screening assessment. The study complied with all relevant ethical regulations and was approved by the London South East NHS Research Ethics Committee (Ref:13/LO/1857). The majority of participants provided their written informed consent to participate, however, the study was also open to patients who lacked the capacity to consent provided that a consultee advised assent on their behalf (see McQueen et al.^69^).

### Clinical interviews

Clinical information and MRI scans were acquired at baseline (14–0 days before commencing clozapine), and repeated 12 weeks after clozapine initiation. Information was obtained via self-report, through review of medical records, and from the treating clinician. Symptom severity was primarily assessed using the Positive and Negative Syndrome Scale (PANSS)^100^ and functioning was assessed using the Global Assessment of Functioning scale (GAF)^101^. Plasma clozapine levels were measured at 6 and 12 weeks. In cases where clozapine levels were below the therapeutic threshold of 350 ng/mL^52^ at 6 weeks, an additional measurement was taken at 8 weeks.

### MRI data acquisition

Magnetic resonance imaging data were acquired on a 3 Tesla MR750 scanner (General Electric, Chicago, USA), which undergoes regular quality control checks including assessment of image contrast, artifacts, and geometry. The scanning session commenced with a localiser, standard axial T2-weighted fast spin echo, and FLAIR scan. T1-weighted images were acquired in a sagittal plane (slice thickness: 1.2 mm, number of slices: 196, field of view 270 mm) using a three-dimensional T1-weighted inversion recovery spoiled gradient-echo (IR-SPGR) Alzheimer’s Disease Neuroimaging Initiative (ADNI-GO, http://adni.loni.usc.edu/) sequence (repetition time: 7.31 ms; echo time: 3.02 ms, inversion time 400 ms, flip angle 11°, acquisition matrix 256 × 256 × 200). ^1^H-MRS data were acquired in 2 × 2 × 2 cm voxels located in the ACC and right caudate nucleus, using point resolved spectroscopy (PRESS) ^69^.

### MRI data processing

T1-weighted images were automatically processed using the longitudinal stream in Freesurfer (https://surfer.nmr.mgh.harvard.edu/), version 6.0.0^97,102,103^. This method creates an unbiased template from both timepoints in each subject for subsequent processing steps, including Desikan-Killiany Atlas segmentation and surface reconstruction. By incorporating the within-subjects template, this method increases reliability and statistical power, making it suitable for small sample sizes^97^. Quality control followed the ENIGMA consortium protocols (http://enigma.ini.usc.edu/) for subcortical volumes and cortical thickness. In one patient, data were excluded from all analyses due to QC failure. No individual brain structures were excluded from analyses due to QC failure. Concentrations of glutamate and NAA (measured as the sum of N-acetylaspartate and N-acetylaspartylglutamate) were estimated using LCModel version 6.3-0I^104^ and reviewed for QC^69^. Metabolite concentration estimates were corrected for voxel tissue composition, termed Glu_corr_ and NAA_corr_^69^.

### Statistical analysis

Changes in subcortical volume and cortical thickness over 12 weeks of clozapine treatment were evaluated as the symmetrized percentage change (SPC). SPC is the rate of change, in mm^3^/year for volume and mm/year for thickness, with respect to the average value for both timepoints. The rate of change was calculated as 100 * (follow-up value − baseline value)/0.23 years and the average value was calculated as 0.5 * (baseline value + follow-up value). This approach, therefore, simplifies longitudinal data in each subject to a single statistic and is suitable for evaluating linear relationships across two timepoints. As SPC calculates the rate with respect to the average value it is more robust than calculating the percentage change with respect to a single baseline timepoint ^97^.

Statistical analysis of extracted subcortical volumes was performed in the Statistical Package for Social Sciences (SPSS Inc., v26, IBM, New York, USA). Caudate, putamen, thalamus, hippocampus, and lateral ventricle volumes in each hemisphere were summed to give a single value for the primary analyses. Shapiro–Wilk tests were used to test for normality of distribution. One sample t-tests or Wilcoxon signed rank tests evaluated whether subcortical volumetric change (SPC) significantly differed from zero. The threshold for statistical significance for the five subcortical volumes had a Bonferroni corrected threshold of α = 0.01. Subsequent sensitivity analyses excluded any outlying values, identified as those 1.5 × the interquartile range (IQR) above the 3rd quartile (Q3) or below the 1st quartile (Q1).

Preparation of cortical data included smoothing (15 mm full width at half maximum) and mapping the images to the Freesurfer average subject image, performed using the Freesurfer long_mris_slopes command which includes calculation of SPC. GLM (mri_glmfit) evaluated whether the SPC in cortical thickness during clozapine treatment differed significantly from zero. Cluster-wise correction for multiple comparisons was performed using simulation of 1000 random permutations, a cluster forming threshold of *P* < 0.05, and cluster-wise *P* < 0.05, adjusted for two hemispheres^105^. Significant clusters were visualised using TKSurfer.

Pearson’s or Spearman’s correlation coefficients examined relationships between structural variables and eTIV, age, plasma clozapine levels at 12 weeks, and percentage change in PANSS Total scores. The percentage change in PANSS Total score was calculated as ((follow up value − baseline value)/baseline value * 100). Minimum possible PANSS scores were subtracted before the calculation of the percentage change^106^. Potential effects of sex were determined using T-tests or Mann–Witney U-tests. Associations between right caudate volume SPC and right caudate NAA_corr_ and Glu_corr_ SPC, and SPC extracted from significant areas of cortical thinning and SPC in ACC NAA_corr_ and Glu_corr,_ were evaluated using Pearson’s or Spearman’s correlation coefficient, with Bonferroni correction (0.05/number of comparisons). Subsequent sensitivity analyses excluded any outlying values, identified as those with a Cook’s D of greater than 3 times the mean.

### Reporting summary

Further information on research design is available in the Nature Research Reporting Summary linked to this article.

## Supplementary information

REPORTING SUMMARY

Supplemental Material

## Data Availability

This study supports data sharing, in line with MRC policy. To apply for access to the anonymized study data, please contact Alice.Egerton@kcl.ac.uk.
