# Supplementary material for: Subcortical volume reduction and cortical thinning 3 months after switching to clozapine in treatment resistant schizophrenia
Source: Schizophrenia (Heidelb). 2022 Mar 2;8(1):13. doi: 10.1038/s41537-022-00230-2 (PMC8891256; doi:10.1038/s41537-022-00230-2)
Supplement: Supplementary file 1 — REPORTING SUMMARY [file 41537_2022_230_MOESM1_ESM.pdf]

nature portfolio

Corresponding author(s): Professor Alice Egerton

Last updated by author(s): 20 Nov 2021

Reporting Summary

Nature Portfolio wishes to improve the reproducibility of the work that we publish. This form provides structure for consistency and transparency in reporting. For further information on Nature Portfolio policies, see our Editorial Policies and the Editorial Policy Checklist.

Statistics

For all statistical analyses, confirm that the following items are present in the figure legend, table legend, main text, or Methods section.

n/a	Confirmed	
☐	☒	The exact sample size (n) for each experimental group/condition, given as a discrete number and unit of measurement
☐	☒	A statement on whether measurements were taken from distinct samples or whether the same sample was measured repeatedly
☐	☒	The statistical test(s) used AND whether they are one- or two-sided<br>*Only common tests should be described solely by name; describe more complex techniques in the Methods section.*
☐	☒	A description of all covariates tested
☐	☒	A description of any assumptions or corrections, such as tests of normality and adjustment for multiple comparisons
☐	☒	A full description of the statistical parameters including central tendency (e.g. means) or other basic estimates (e.g. regression coefficient) AND variation (e.g. standard deviation) or associated estimates of uncertainty (e.g. confidence intervals)
☐	☒	For null hypothesis testing, the test statistic (e.g. F, t, r) with confidence intervals, effect sizes, degrees of freedom and P value noted<br>*Give P values as exact values whenever suitable.*
☒	☐	For Bayesian analysis, information on the choice of priors and Markov chain Monte Carlo settings
☐	☒	For hierarchical and complex designs, identification of the appropriate level for tests and full reporting of outcomes
☐	☒	Estimates of effect sizes (e.g. Cohen's d, Pearson's r), indicating how they were calculated

Our web collection on statistics for biologists contains articles on many of the points above.

Software and code

Policy information about availability of computer code

Data collection	Not applicable
Data analysis	T1-weighted images were automatically processed using the longitudinal stream in Freesurfer (https://surfer.nmr.mgh.harvard.edu/), version 6.0.0. Cortical data were prepared using the Freesurfer long_mris_slopes command<br>Concentrations of glutamate and NAA (measured as the sum of N-acetylaspartate and N-acetylaspartylglutamate) were estimated using LCModel version 6.3-0I.<br>Statistical analysis was conducted in SPSS, version 26 and using Freesurfer mri_glmfit.

For manuscripts utilizing custom algorithms or software that are central to the research but not yet described in published literature, software must be made available to editors and reviewers. We strongly encourage code deposition in a community repository (e.g. GitHub). See the Nature Portfolio guidelines for submitting code & software for further information.

Data

Policy information about availability of data

All manuscripts must include a data availability statement. This statement should provide the following information, where applicable:

- Accession codes, unique identifiers, or web links for publicly available datasets
- A description of any restrictions on data availability
- For clinical datasets or third party data, please ensure that the statement adheres to our policy

This study supports data sharing, in line with MRC policy. To apply for access to the anonymized study data, please contact Alice.Egerton@kcl.ac.uk

Field-specific reporting

Please select the one below that is the best fit for your research. If you are not sure, read the appropriate sections before making your selection.

☒ Life sciences ☐ Behavioural & social sciences ☐ Ecological, evolutionary & environmental sciences

For a reference copy of the document with all sections, see nature.com/documents/nr-reporting-summary-flat.pdf

Life sciences study design

All studies must disclose on these points even when the disclosure is negative.

Sample size	The sample size was determined on the basis of data presented in our previous manuscript (McQueen et al., Schizophrenia Bulletin, 47, 662-671, 2021)
Data exclusions	Quality control followed the ENIGMA consortium protocols (http://enigma.ini.usc.edu/) for subcortical volumes and cortical thickness. In one patient, data were excluded from all analyses due to QC failure. No individual brain structures were excluded from analyses due to QC failure.
Replication	The study did not include replication. The results are broadly consistent with previous literature
Randomization	Not relevant as there was only one group of participants
Blinding	Not relevant as there was only one group of participants

Reporting for specific materials, systems and methods

We require information from authors about some types of materials, experimental systems and methods used in many studies. Here, indicate whether each material, system or method listed is relevant to your study. If you are not sure if a list item applies to your research, read the appropriate section before selecting a response.

Materials & experimental systems

n/a	Involved in the study	
☒	☐	Antibodies
☒	☐	Eukaryotic cell lines
☒	☐	Palaeontology and archaeology
☒	☐	Animals and other organisms
☐	☒	Human research participants
☒	☐	Clinical data
☒	☐	Dual use research of concern

Methods

n/a	Involved in the study	
☒	☐	ChIP-seq
☒	☐	Flow cytometry
☐	☒	MRI-based neuroimaging

Human research participants

Policy information about studies involving human research participants

Population characteristics	Patients with an ICD-10 diagnosis of schizophrenia or schizoaffective disorder, the presence of treatment resistant illness, and being about to commence clozapine titration as part of normal clinical care. Presence of TRS was inferred from medical records and discussion with the treating psychiatrist, with criteria including at least two previous trials of a non-clozapine antipsychotic within the recommended dose range for at least 6 weeks and referral for clozapine initiation.<br>Age mean ± s.d. 38.62 ± 12.81 years; sex 18 male 6 female.
Recruitment	Participants were recruited from inpatient and outpatient services within the South London and Maudsley and tOxleas NHS Foundation Trusts. INCLUSION: meeting ICD-10 criteria for schizophrenia (F20) or schizoaffective disorder (F25), as diagnosed by their treating psychiatrist. Being due to switch from their current antipsychotic to clozapine as part of their normal clinical care; Participants with mental capacity to consent provided written informed consent to study procedures. The study was also open to participants lacking capacity to consent if a consultee advised assent on their behalf. The consultee was defined as a person nonprofessionally involved in caring for the patient or concerned with their welfare (normally their next-of-kin family member). They were advised on the role of the consultee, provided with a consultee information sheet and invited to ask any questions about the study before advising on the patient's behalf. If there was any indication from the patient, consultee, clinical team, or anyone else involved in the patient's care that the patient would not wish to participate, they were not enrolled in the study. During study participation, study researchers maintained contact with the consultee and clinical team. If the patient expressed any objections to the study or wishes to withdraw, they were withdrawn from the study immediately. General exclusion criteria included drug dependency, pregnancy and contraindications to MRI.
Ethics oversight	London South East NHS ethics committee (Ref:13/LO/1857).

Note that full information on the approval of the study protocol must also be provided in the manuscript.

Magnetic resonance imaging

Experimental design

Design type	Structural T1-weighted MRI
Design specifications	Not applicable
Behavioral performance measures	Not applucable

Acquisition

Imaging type(s)	Structural
Field strength	3 Tesla
Sequence & imaging parameters	T1-weighted images were acquired in a sagittal plane (slice thickness: 1.2mm, number of slices: 196, field of view 270mm) using a 3-dimensional T1-weighted inversion recovery spoiled gradient-echo (IR-SPGR) Alzheimer's Disease Neuroimaging Initiative (ADNI-GO, http://adni.loni.usc.edu/) sequence (repetition time: 7.31ms; echo time: 3.02ms, inversion time 400ms, flip angle 11°, acquisition matrix 256 x 256 x 200).
Area of acquisition	whole brain

Diffusion MRI ☐ Used ☒ Not used

Preprocessing

Preprocessing software	automatic processing using the longitudinal stream in Freesurfer (https://surfer.nmr.mgh.harvard.edu/), version 6.0.0.82-84 This method creates an unbiased template from both timepoints in each subject for subsequent processing steps, including Desikan-Killiany Atlas segmentation and surface reconstruction. Preparation of cortical data included smoothing (15mm full width at half maximum) and mapping the images to the Freesurfer average subject image, performed using the Freesurfer long_mris_slopes command.
Normalization	see above
Normalization template	see above
Noise and artifact removal	not applicable
Volume censoring	not applicable

Statistical modeling & inference

Model type and settings	one sample t-test, GLM or wilcoxon signed rank tests.
Effect(s) tested	whether symmetrized percentage change in subcortical volume or cortical thickness differed significantly from zero

Specify type of analysis: ☐ Whole brain ☐ ROI-based ☒ Both

Anatomical location(s): Desikan-Killiany Atlas

Statistic type for inference (See Eklund et al. 2016)	ROI mean value and Cluster-wise
Correction	ROI: Bonferroni correction. Cluster-wise: simulation of 1000 random permutations, a cluster forming threshold of P < 0.05 and cluster-wise P < 0.05, adjusted for two hemispheres

Models & analysis

n/a	Involved in the study
☒	☐ Functional and/or effective connectivity
☒	☐ Graph analysis
☒	☐ Multivariate modeling or predictive analysis
