# Supplementary material for: Subcortical volume reduction and cortical thinning 3 months after switching to clozapine in treatment resistant schizophrenia
Source: Schizophrenia (Heidelb). 2022 Mar 2;8(1):13. doi: 10.1038/s41537-022-00230-2 (PMC8891256; doi:10.1038/s41537-022-00230-2)
Supplement: Supplementary file 2 — Supplemental Material [file 41537_2022_230_MOESM2_ESM.pdf]

Supplementary Material: Subcortical volume reduction and cortical thinning after switching clozapine in treatment resistant schizophrenia.

**Cortical cluster**	**Subcortical volume**	**Statistic**
Left inferior temporal	Caudate	df =21; r = 0.15; P = 0.50
	Putamen	df =21; r = 0.30; P = 0.17
Left caudal middle frontal	Caudate	df =21; r = 0.26; P = 0.23
	Putamen	df =21; r = 0.26; P = 0.23
Right temporal pole	Caudate	df =21; r = 0.31; P = 0.15
	Putamen	df =21; r = 0.61; P = 0.002

Supplement Table 1. Relationships between symmetrized percentage change (SPC) in cortical thickness and SPC in caudate and putamen volume over 12 weeks of clozapine treatment in TRS, corrected for sex.

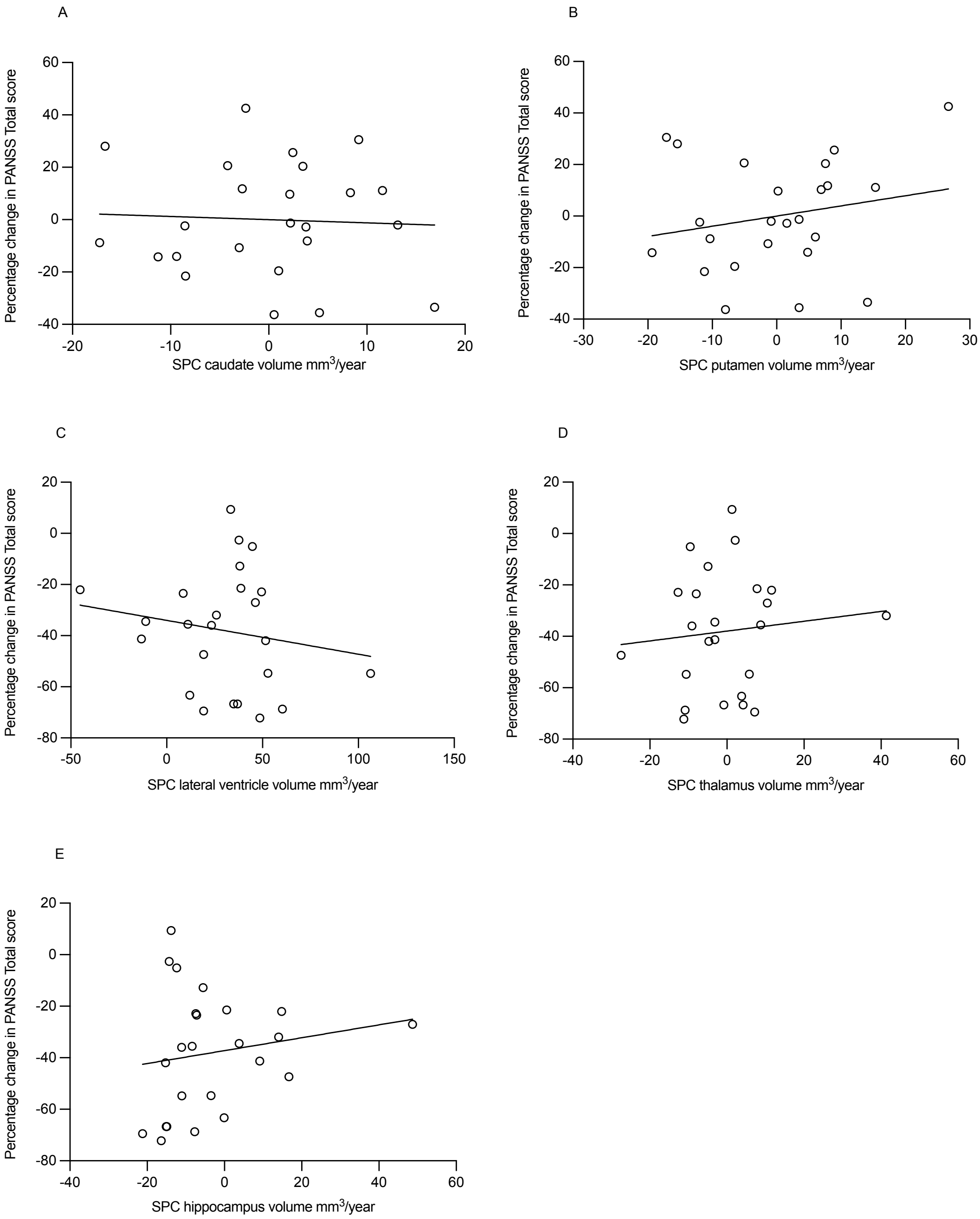

Supplement Figure 1. Relationships between subcortical symmetrized percentage change (SPC) in subcortical volume and the percentage change in Positive and Negative Syndrome Scale for Schizophrenia (PANSS) Total scores over 12 weeks of clozapine treatment in TRS. Partial plots for the caudate (A) and putamen (B) individual data points corrected for sex. Plots for the lateral ventricles (C), thalamus (D) and hippocampus (E) present uncorrected individual values. None of the relationships between SPC and percentage change PANSS Total score were statistically significant (all $P > 0.05$).

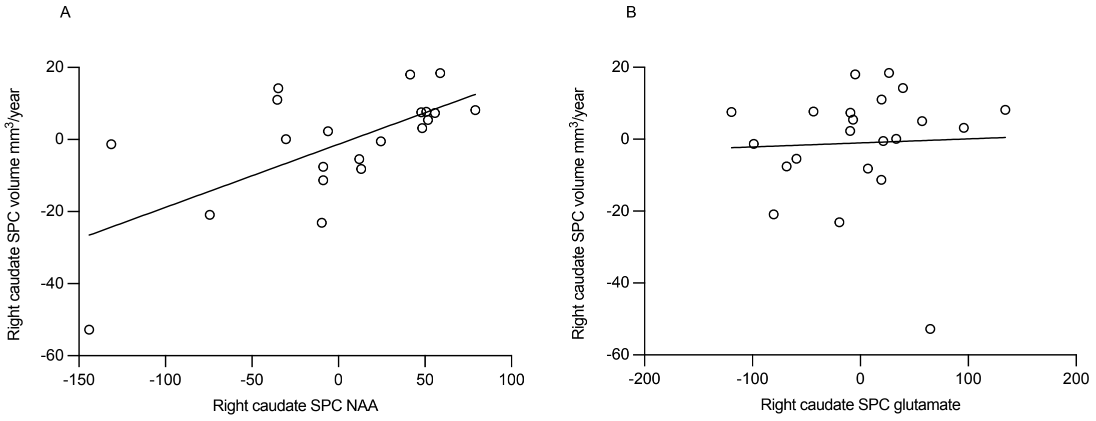

Supplement Figure 2. Partial correlation plots of the symmetrized percentage change in NAA (A) and glutamate (B) and volume of the right caudate nucleus over 12 weeks of clozapine treatment. Data are adjusted for effects of sex. The relationship between SPC in right caudate NAA and SPC in right caudate volume was significant (df = 18, r = 0.62; *P* = 0.002). The relationship between SPC in glutamate and caudate volume was non-significant (*P* > 0.05). NAA: N-acetylaspartate; SPC: symmetrized percentage change.
